# ﻿Five new species of the genus *Grouvellinus* Champion, 1923 from Guizhou Province, China (Coleoptera, Elmidae)

**DOI:** 10.3897/zookeys.1219.125754

**Published:** 2024-11-27

**Authors:** Ri-Xin Jiang, Ping-Lan Wu, Xiang-Sheng Chen

**Affiliations:** 1 Institute of Entomology, Guizhou University, Guiyang 550025, Guizhou, China; 2 The Provincial Special Key Laboratory for Development and Utilization of Insect Resources of Guizhou, Guizhou University, Guiyang, 550025, Guizhou, China; 3 The Provincial Key Laboratory for Agricultural Pest Management of Mountainous Region, Guiyang 550025, Guizhou, China; 4 Agricultural Service Center of Yanlou Town, Huaxi District, Guiyang 550025, Guizhou, China

**Keywords:** Aquatic beetle, Elminae, riffle beetles, Southwestern China, water environment

## Abstract

The genus *Grouvellinus* Champion, 1923 comprises 60 described species distributed across the Oriental and Palearctic regions. Species diversity is very high in mainland China, with 28 recorded species. Here, the results of the aquatic beetle survey in Guizhou Province are presented; they began in 2021, when we collected more than 5000 specimens of riffle beetle. All specimens come from small ravine streams where inhabited submerged stones. Using morphological characters of adults such as body form and size, coloration, elytral, pronotal and ventral surface structures, and forms of male and female genitalia, five new species were discovered and are described: *Grouvellinusloong***sp. nov.**, *G.buyi***sp. nov.**, *G.wangmoensis***sp. nov.**, *G.lihaitaoi***sp. nov.**, *G.muyinlini***sp. nov.** The species descriptions contain illustrations of diagnostic characters and measurements of metric characters such as body length, pronotal length, pronotal width, elytral length and width, and head length and width. The females are, on average, larger and have only slight differences in external morphology compared to the males. The comparative diagnoses discuss characters of the new and already known species. The results show that the existing species diversity requires more detailed research focusing on larger areas of South China in the future.

## ﻿Introduction

The aquatic beetle family Elmidae (also known as riffle beetles) has a worldwide distribution with its highest diversity in the tropics (Kodada et al. 2016). Members of this family are sometimes used as indicators for freshwater quality (Moog and Jäch 2003; Junqueira et al. 2010). Riffle beetles are usually found in undisturbed streams, where they dwell in benthic microhabitats and feed on algae and detritus (Kodada et al. 2016).

The genus *Grouvellinus* Champion, 1923 is widely distributed in the Oriental and Palearctic regions, and has included 60 known species (Jung et al. 2014; Bian and Sun 2016; Jäch et al. 2016; Bian and Jäch 2018, 2019; Freitag et al. 2018, 2020; Bian and Zhang 2023; Jiang et al. 2023). This genus exhibits a high diversity in China, with 28 species recorded from mainland China (Jäch and Kodada 1995; Bian and Zhang 2023; Jiang et al. 2023). The Chinese fauna of this genus was recently reviewed by a series of papers, and many new species were described (Bian and Sun 2016; Bian and Jäch 2018, 2019; Bian and Zhang 2023; Jiang et al. 2023). Previously to this work, there was only one *Grouvellinus* species in Guizhou Province: *Grouvellinushuaxiensis* Jiang, Huang & Chen, 2023, found in an urban river of Guiyang City (Jiang et al. 2023).

Through our aquatic beetle survey in Guizhou Province, which began in 2021, a large number (more than 5000 specimens) of riffle beetles were collected, many of them belonging to the genus *Grouvellinus*. In the present paper, five new species of this genus are described and illustrated. A distribution map (Fig. 13) of the known *Grouvellinus* species from Guizhou Province is also provided.

## ﻿Materials and methods

The examined material is deposited in the Institute of Entomology, Guizhou University, Guiyang, China (**GUGC**). Label data are quoted verbatim. The Chinese translation of each locality below the provincial level is included in parentheses at its first appearance in the text. Each type specimen bears the following label: ‘HOLOTYPE (red) (or PARATYPE (yellow)), m# (or f#), *Grouvellinus* + specific name sp. nov., Jiang, Wu & Chen, 2024.’

Dissected parts were preserved in Euparal on plastic slides and were placed on the same pin with the specimen. Habitus images were taken using a Canon 5D Mark IV digital camera with MP-E 65 mm f/2.8 1–5× macro lens. A Godox MF12 flash was used as the light source. Images of the morphological structures were taken using a Canon 5D Mark IV digital camera with a Mitutoyo Plan NIR 10 lens and a Godox MF12 flash was used as the light source, or a Nikon SMZ25 stereoscopic microscope with a Nikon DS-Ri2 camera. Genitalia were soaked in 10% NaOH solution for half an hour, and dehydrated in 100% alcohol, then preserved in Euparal on plastic slides. Images of genitalia were taken using a Nikon N1-E microscope with a Nikon DS-Ri2 camera. Zerene Stacker v. 1.04 was used for image stacking. All images were modified and grouped into plates using Adobe Photoshop CS5 Extended. Measurements of metric characters were taken by NIS-Elements AR, and added in Microsoft Excel to compute mean and standard deviations.

Morphological terminology and the format for the descriptions follow those of Bian and Sun (2016). The following abbreviations are used: **HL**—length of head from the anterior clypeal margin to the occipital constriction; **HW**—width of head across eyes; **PL**—length of pronotum along the midline; **PW**—maximum width of pronotum; **EL**—length of elytra along the suture; **EW**—maximum width of elytra; **CL**—length of body, as the sum PL + EL.

## ﻿Taxonomy

### ﻿ Grouvellinus

Taxon classificationAnimaliaColeopteraElmidae

Champion, 1923

BEAB2A2B-7F82-50DF-9344-48F8430605A7

Grouvellinus Champion, 1923: 168. Type species: Macronychuscaucasicus Victor, 1839.

### ﻿ Grouvellinus loong sp. nov.

Taxon classificationAnimaliaColeopteraElmidae

B2475873-489A-5D94-B78B-CCF07AE3771A

https://zoobank.org/134104C8-B9C1-4B30-BCF1-B009441214C4

**Figure 1.**
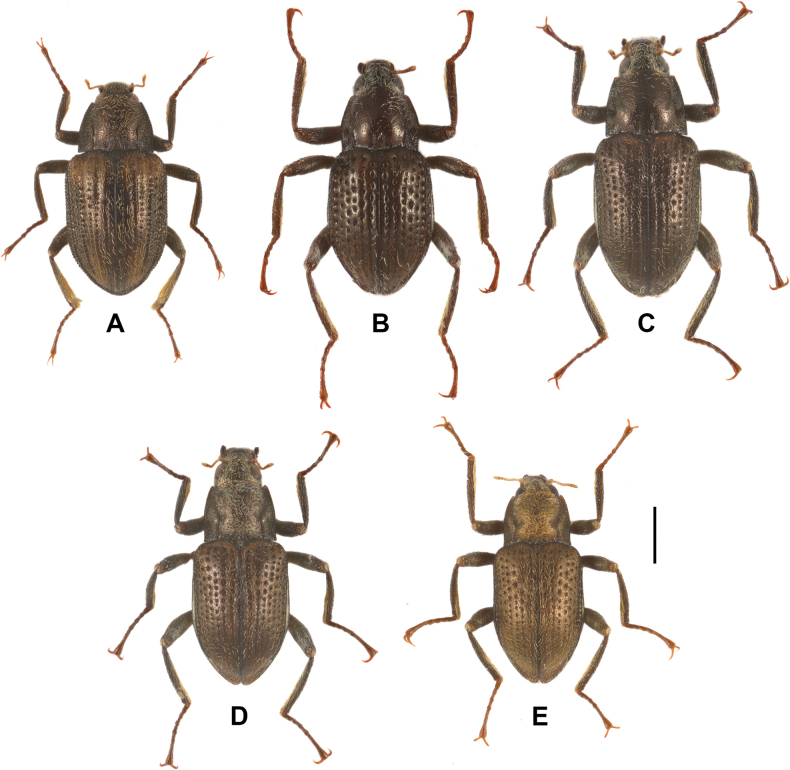
Dorsal habitus of *Grouvellinus* species, holotypes **A***G.loong* sp. nov. **B***G.buyi* sp. nov. **C***G.wangmoensis* sp. nov. **D***G.lihaitaoi* sp. nov. **E***G.muyinlini* sp. nov. Scale bar: 0.5 mm.

**Figure 2.**
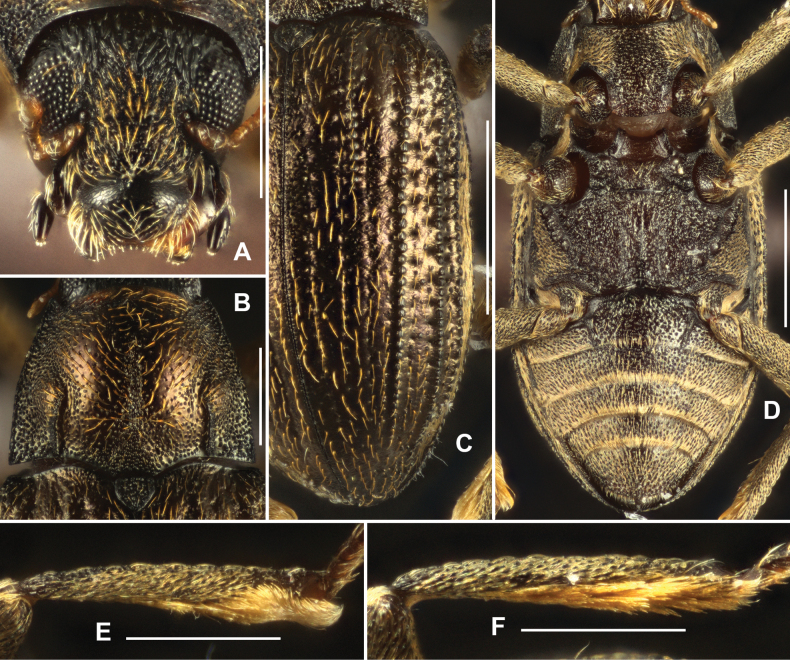
Diagnostic features of *Grouvellinusloong* sp. nov., paratype **A** head, dorsal view **B** pronotum, dorsal view **C** elytra, dorsal view **D** ventral habitus **E** metatibia, male **F** same, female. Scale bars: 0.25 mm (**A, B, E, F**); 0.5 mm (**C, D**).

**Figure 3.**
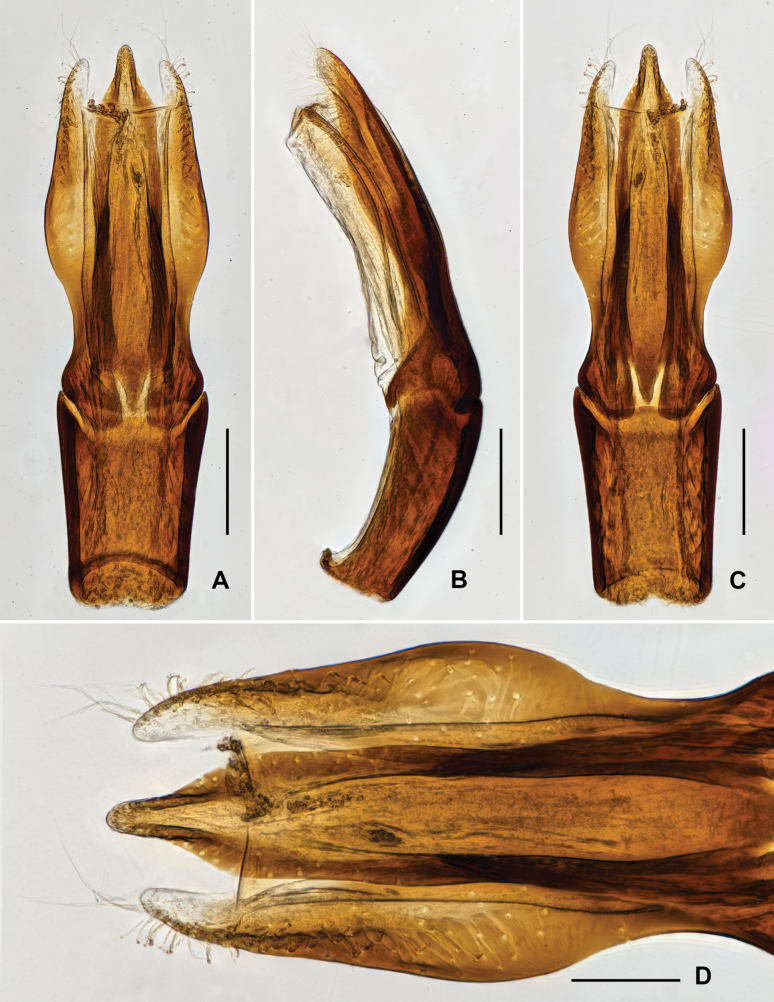
Aedeagus of *Grouvellinusloong* sp. nov., paratype **A** ventral view **B** lateral view **C** dorsal view **D** apex of median lobe and parameres. Scale bars: 0.05 mm (**D**); 0.1 mm (**A–C**).

Figs 1A , 2 , 3 , 12A, F

#### Material examined.

(42 exs: 21 ♂♂, 21 ♀♀): ***Holotype*: China** • ♂, labeled ‘China: Guizhou, Qiannan Buyi and Miao Autonomous Prefecture (黔南布依族苗族自治州), Luodian County (罗甸县), Luokun Town (罗悃镇), Xiangshui Village (响水村), 25°19'43"N, 106°38'28"E, H: 666.10±6.40m, 09.XI.2022, Jiang Ri-Xin leg.’ (GUGC). ***Paratypes*: China** • 20 ♂♂, 21 ♀♀, with same label data as the holotype (GUGC).

#### Diagnosis.

Body obovate; elytra dark brown, with weak cupreous metallic luster, each elytron with yellowish brown spot at base and near apex. Elytral intervals III, V, VII, and VIII carinated; carinae granulated, those on interval III short, extending from base to ~ 1/3 of elytral length; other carinae long and extending from base nearly to elytral apex. Lateral margins of elytra granulated. Aedeagus with median lobe constricted near middle and in apical 1/3; apex narrowed, subacute. Parameres nearly as long as median lobes, strongly narrowed at basal 1/3, apex rounded, lateral portion with long setae at apical 1/3.

#### Description.

**Male.** Body obovate (Fig. 1A); head, pronotum and elytra black or dark brown, with weak cupreous metallic luster; femora and tibiae black; tarsi and antenna reddish brown; elytra with four reddish brown spots. Plastron area confined to head except for frons, vertex, and clypeus, lateral portions of prosternum, ninth elytral interval and epipleura, lateral portions of mesosternum, metaventrite, abdomen, femora, and tibia.

Head (Fig. 2A) wider than long, dorsal surface with dense short setae (except discal part) and large round punctures, longer setae sparsely and finely located at dorsal surface. Clypeus evenly punctate with large round punctures and with sparse long setae. Labrum transverse, slightly narrower than clypeus, surface distinctly microreticulated, apical 1/2 covered with sparse long setae, lateral margins with long bristles, apical margin rounded.

Pronotum (Fig. 2B) wider than long, widest at base, narrowed anteriad. Disc sparsely punctured on admesal portion, punctures with longer adpressed setae, intervals glabrous, shiny; longitudinal mesal portion with dense small punctures along posterior 2/3, impression absent; surface densely granulated. Anterior margin arcuate, anterior angles distinctly produced and acutangular with subacute apex; pronotal margins finely curved. Basal margin trisinuate, posterior angles acutangular, with apex rounded. Sublateral carinae distinct, extending from base to ~ 1/2 length of pronotum; basal 1/2 straight, near parallel; apical 1/2 curved; each side of sublateral carinae with a shallow and indistinct oblique impression, extending from apical end of carina to near anterior angle. Prosternal process (Fig. 2D) with weakly curved apex, surface densely covered with large punctures.

Scutellum (Fig. 2B) subtriangular, wider than long, widest near middle; surface covered with large punctures and each puncture bearing a short seta; lateral margins finely curved, apex obtuse.

Elytra (Fig. 2C) > 1.5 × as long as wide, widest at base, narrowed to apex. Granulate carinae on intervals III, V, VII, and VIII; carina on interval III short, ~ 1/3 length of elytra; other carinae long, extending from base of elytra to near apex. Lateral margins of elytra granulated. Each elytron with two reddish brown spots, one at base and another one near apex.

Metaventrite (Fig. 2D), surface rough with sparse long setae and large round punctures at disc. Median sulcus long but indistinct, extending from posterior margin to anterior 1/4, basal 1/4 wide and deep, apical 3/4 thin and shallow. Lateral sides of disc with series of elliptical shallow impression.

***Abdomen*.** Middle regions of ventrites I–IV and anterior part of ventrite V punctured (Fig. 2D), covered with sparse large round punctures, each puncture bearing a long seta. Other parts of abdomen (Fig. 2D) covered with plastron and mixed with sparse long setae. Apex of ventrite V rounded. Ventrite I with well-developed pair of admedian carinae, straight, extending from base to apex.

Legs simple, surface granulated; femora widened, surface with plastron; inner side of tibiae with cleaning fringes; metatibiae (Fig. 2E) distinctly narrowed near apex; tarsi slightly shorter tibiae; tarsal claws simple.

***Genitalia*.** Aedeagus (Fig. 3A–D), long and wide, symmetrical; median lobe slightly surpasses parameres, narrowed near middle and in apical 1/3, apex narrowed, subacute. Parameres (Fig. 3D) strongly narrowed at basal 1/3, with apical portion narrowed; apical 2/5 covered with long setae.

**Female.** Externally similar to the male, averagely larger, metatibiae (Fig. 2F) not narrowed near apex. Ovipositor as in Figs 12A, F. Stylus short and narrow, weakly curved, ~ 1/6 as long as distal portion of coxite. Coxite long, with apex distinctly expanded, roundly broadened at outer margin, apex with several short and curved sensilla. Distal portion of coxite nearly straight, surface with sparse, very short and acute setae. Proximal portion of coxite short, longer than 1/3 length of distal portion, surface with sparse, very short and acute setae. Valvifers slightly shorter than coxite, longitudinal baculum curved.

#### Measurements.

**Male** (*n* = 10): CL: 1.81–1.89 mm (1.85±0.03); PL: 0.51–0.54 mm (0.52±0.01), PW: 0.68–0.74 mm (0.70±0.02); EL: 1.30–1.35 mm (1.33±0.02), EW: 0.90–0.93 mm (0.91±0.01).

**Female** (*n* = 10): CL: 1.68–2.08 mm (1.90±0.12); PL: 0.49–0.63 mm (0.56±0.05), PW: 0.62–0.80 mm (0.71±0.06); EL: 1.20–1.45 mm (1.35±0.09), EW: 0.86–0.96 mm (0.91±0.04).

#### Distribution.

China (Guizhou).

#### Biology.

All adults were collected from submerged stone in small ravine stream (Fig. 14A–C).

#### Etymology.

The specific epithet ‘loong’ is the most famous auspicious beast in Chinese ancient myth, and also is a member of the Chinese Zodiac Signs.

#### Comparative diagnosis.

The new species can be placed in the *Grouvellinusacutus* species group by the following characters: 1) body small; 2) elytra with yellowish brown markings; 3) pronotum without longitudinal impression; 4) surface of elytra with granulate carinae on strial intervals III, V, VII, and VIII; 5) ventrite I with a pair of well-developed admedian carinae. The *G.acutus* species group includes three known species, all of them occurring in China. Members of this group are similar in habitus. *G.loong* sp. nov. can be easily distinguished from other members of this group by the obviously different shape of the aedeagus: strongly narrowed at middle (vs not as above).

*Grouvellinusloong* sp. nov. is most similar to *G.acutus* Bian & Jäch, 2018 in habitus. These species can be distinguished by the following characters: 1) prosternal process with weakly curved apex, surface not granulated, densely covered with large punctures in *G.loong* sp. nov. (vs prosternal process with broadly rounded apex, surface sparsely granulated in *G.acutus*); 2) metatibia distinctly narrowed near apex, without hooked appendage at apex in *G.loong* sp. nov. (vs apex of metatibia conspicuously broad and sclerotized, usually with hooked appendage in *G.acutus*); 3) parameres of aedeagus strongly narrowed at basal 1/3 in *G.loong* sp. nov. (vs only weakly narrowed near base in *G.acutus*).

### ﻿ Grouvellinus buyi sp. nov.

Taxon classificationAnimaliaColeopteraElmidae

10E10C38-6330-5894-B68C-AFEFF132E206

https://zoobank.org/8C824860-8A11-4910-BA45-830E72C9D67A

**Figure 4.**
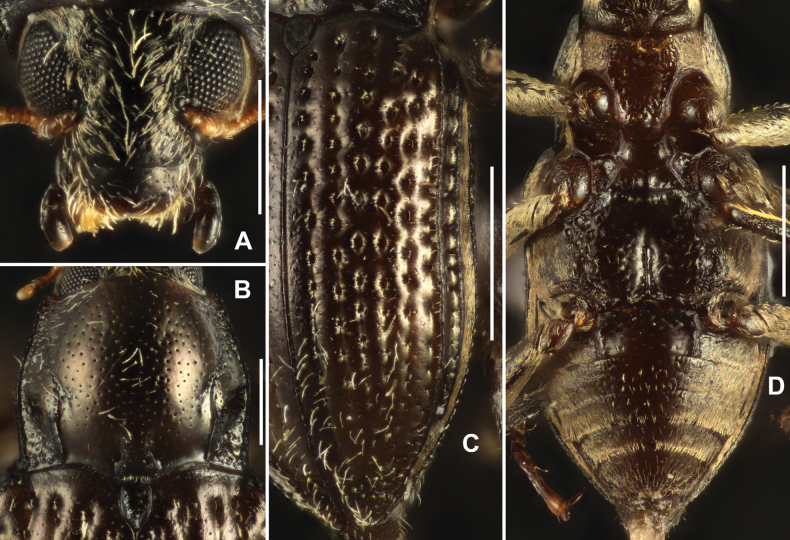
Diagnostic features of *Grouvellinusbuyi* sp. nov., paratype **A** head, dorsal view **B** pronotum, dorsal view **C** elytra, dorsal view **D** ventral habitus. Scale bars: 0.25 mm (**A, B**); 0.5 mm (**C, D**).

**Figure 5.**
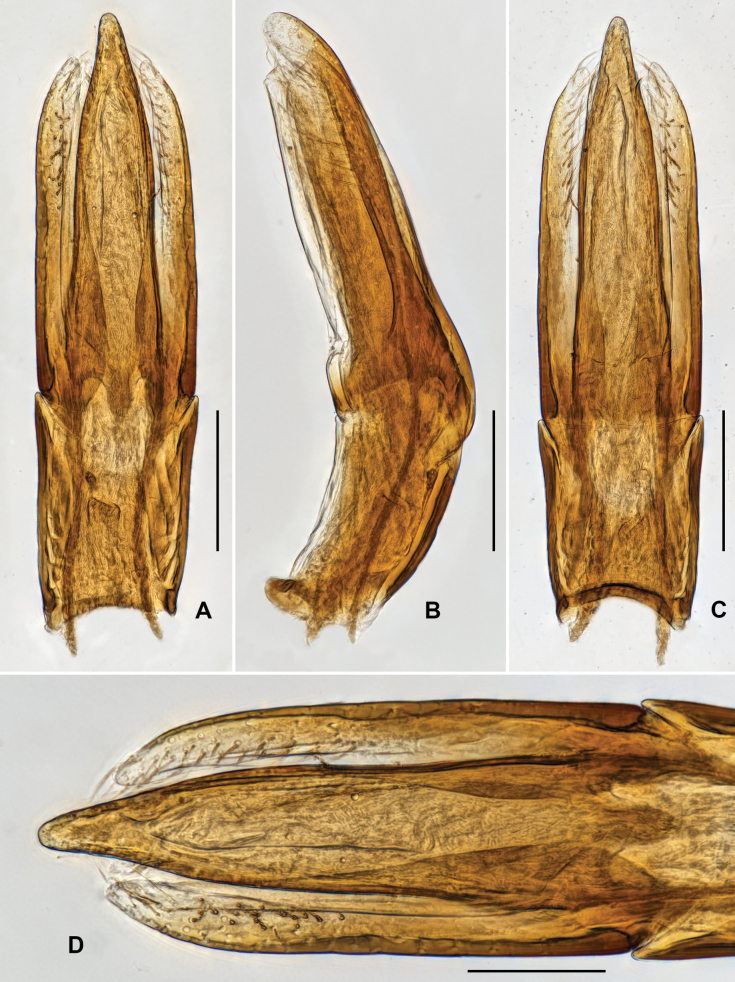
Aedeagus of *Grouvellinusbuyi* sp. nov., paratype **A** dorsal view **B** lateral view **C** ventral view **D** apex of median lobe and parameres. Scale bars: 0.05 mm (**D**); 0.1 mm (**A–C**).

Figs 1B , 4 , 5 , 12B, G

#### Material examined.

(11 exs: 5 ♂♂, 6 ♀♀): ***Holotype*: China** • ♂, labeled ‘China: Guizhou, Guiyang City (贵阳市), Wudang District (乌当区), Xinbaobuyi Township (新堡布依族乡), Xiangzhigou Scenic Area (香纸沟景区), 26°47'02"N, 106°56'09"E, H: 1187m, 06.XI.2022, Jiang Ri-Xin leg.’ (GUGC). ***Paratypes*: China** • 4 ♂♂, 6 ♀♀, with same label data as the holotype (GUGC).

#### Diagnosis.

Body, long-oval, dark brown, surface shiny with cupreous metallic luster. Pronotum widest at basal 2/5, finely covered with small punctures, anterior and posterior angles densely covered with large punctures. Elytral intervals VII and VIII carinated, carinae granulated. Strial punctures of elytra very large in basal 2/3 and much smaller in apical 1/3. Sides of aedeagus generally subparallel in dorsal and ventral view, with median lobe slightly longer than parameres, distinctly curved in lateral view, apex narrowed and subacute. Parameres with apex rounded, lateral portion with long setae at apical 2/5.

#### Description.

**Male.** Body long-oval (Fig. 1B), dark brown with tibiae and antenna pale brown, surface shiny with distinct cupreous metallic luster. Plastron area confined to head except for frons, vertex and clypeus, lateral portions of prosternum, ninth elytral interval and epipleura, lateral portions of mesosternum, metaventrite, abdomen, and femora.

Head (Fig. 4A) wider than long, dorsal surface shiny, densely with short setae (except discal part) and with sparse large punctures, each bearing a longer seta. Surface of clypeus shiny, with sparse small punctures and long setae. Labrum transverse, narrower than clypeus, surface shiny, with sparse short setae, lateral margins with long bristles, apical margin rounded.

Pronotum (Fig. 4B), wider than long, widest near middle. Disc finely punctured with sparse small punctures, punctures with longer adpressed setae, intervals glabrous, shiny. Surface of anterior and posterior angles densely with large punctures. Anterior margin arcuate, anterior angles distinctly produced and acutangular, pronotal margins finely curved. Basal margin trisinuate, posterior angles acutangular. Longitudinal impression absent, with several pairs of small granules located in front of angles of scutellum. Sublateral carinae present in basal 1/2 of pronotum, trisinuate, each side of sublateral carinae with a shallow and indistinct oblique impression, extending from apical end of carina to near anterior angle. Prosternal process (Fig. 4D) with straight apex, surface sparsely with large punctures.

Scutellum (Fig. 4B) half fusiform, ~ 1.5 × as long as wide, widest near middle; surface microreticulated, with several small punctures; lateral margins finely curved, apex acutangular.

Elytra (Fig. 4C) ~ 1.5 × as long as wide, widest behind basal 1/2. Surface shiny, with distinct cupreous metallic luster and rows of sparse long setae. Strial punctures larger in basal 1/2, separated by ~ 1.5 × their diameters, much smaller and widely separated in other parts of elytra. Granulated carinae on strial intervals VII and VIII, other intervals flat. Hind wings well developed.

Metaventrite (Fig. 4D), surface rough with sparse long setae and large round punctures at disc. Median sulcus distinct, extending from posterior margin to anterior 1/2. A pair of small round impression located at basal sides of median sulcus. Lateral sides of disc with series of elliptical shallow impression.

***Abdomen*.** Middle regions of ventrites I–IV and anterior part of ventrite V punctured (Fig. 4D), covered with sparse small punctures, each puncture bearing a long seta. Other parts of abdomen (Fig. 4D) covered with plastron and mixed with sparse long setae. Apex of ventrite V weakly concaved. Ventrite I with well-developed pair of admedian carinae, straight, extending from base to apex.

Legs simple, surface granulated; femora widened, surface with plastron; inner side of tibiae with cleaning fringes; tarsi slightly shorter tibiae; tarsal claws simple.

***Genitalia*.** Aedeagus (Fig. 5A–D), long and slender, symmetrical; median lobe slightly longer than parameres, finely get narrowed in basal 3/4, distal 1/4 distinctly sharpened. Parameres (Fig. 5D) curved at apex, with apex rounded; apical 1/2 covered with long setae.

**Female.** Externally similar to the male, averagely larger. Ovipositor as in Fig. 12B, G. Stylus short and narrow, weakly curved at apex, ~ 1/7 as long as distal portion of coxite. Coxite long, apex weakly expanded, roundly broadened at outer margin, with several short and curved sensilla. Distal portion of coxite nearly straight, surface finely with very short and acute setae. Proximal portion of coxite short, longer than 1/3 length of distal portion, surface finely covered with sparse, very short and acute setae. Valvifers ~ as long as coxite, longitudinal baculum weakly curved.

#### Measurements.

**Male** (*n* = 5): CL: 1.91–2.09 mm (1.96±0.07); PL: 0.58–0.59 mm (0.58±0.01), PW: 0.71–0.74 mm (0.72±0.01); EL: 1.32–1.50 mm (1.38±0.07), EW: 0.94–1.00 mm (0.97±0.02).

**Female**: CL (*n* = 6): 2.18–2.23 mm (2.21±0.02); PL: 0.62–0.65 mm (0.63±0.01), PW: 0.72–0.83 mm (0.77±0.04); EL: 1.56–1.58 mm (1.57±0.01), EW: 0.97–1.10 mm (1.01±0.05).

#### Distribution.

China: Guizhou.

#### Biology.

All adults were collected from submerged stone in small ravine stream (Fig. 14D).

#### Etymology.

The specific epithet ‘buyi’ is a nation of Chinese, which is the majority nation of the type locality of this new species: Xinbaobuyi Township (Guiyang City, Guizhou Province, China).

#### Comparative diagnosis.

The new species is similar with *Grouvellinussinensis* Grouvelle, 1906 and *G.ligulaceus* Bian & Zhang, 2023 in habitus. *Grouvellinusbuyi* sp. nov. can be easily distinguished from *G.ligulaceus* by the following characters: 1) strial punctures of elytra very large in basal 1/2 (vs much smaller); 2) prosternal process with straight apex (vs weakly rounded); 3) median lobe of aedeagus slender (vs much broader); 4) parameres of aedeagus distinctly curved at apex (vs not as above).

The new species can be distinguished from *G.sinensis* by having a body with metallic luster and the surface of metaventrite and abdominal ventrites shiny, whereas the body lacks metallic luster and the surface of metaventrite and abdominal ventrites is distinctly rough in *G.sinensis*.

### ﻿ Grouvellinus wangmoensis sp. nov.

Taxon classificationAnimaliaColeopteraElmidae

CC680CE8-019B-5A1C-9F17-141A47264173

https://zoobank.org/08E7A5E2-AD72-4799-B81B-B7283F8FD4BE

**Figure 6.**
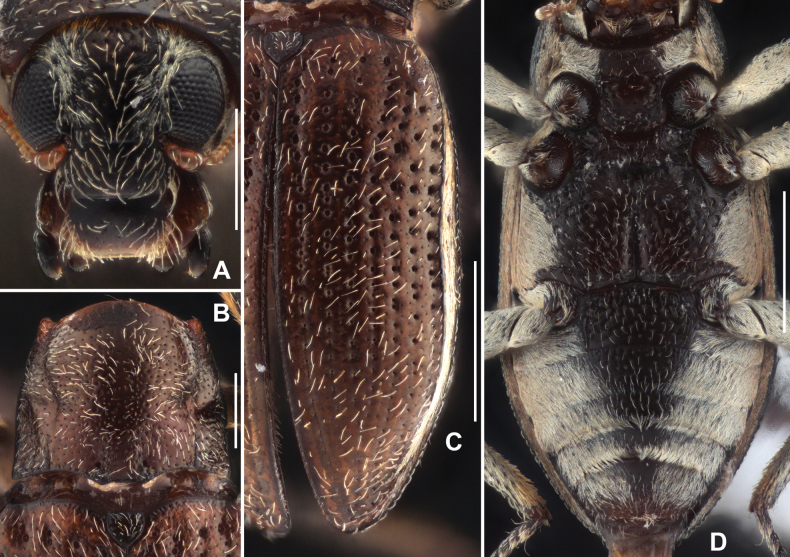
Diagnostic features of *Grouvellinuswangmoensis* sp. nov., paratype **A** head, dorsal view **B** pronotum, dorsal view **C** elytra, dorsal view **D** ventral habitus. Scale bars: 0.25 mm (**A, B**); 0.5 mm (**C, D**).

**Figure 7.**
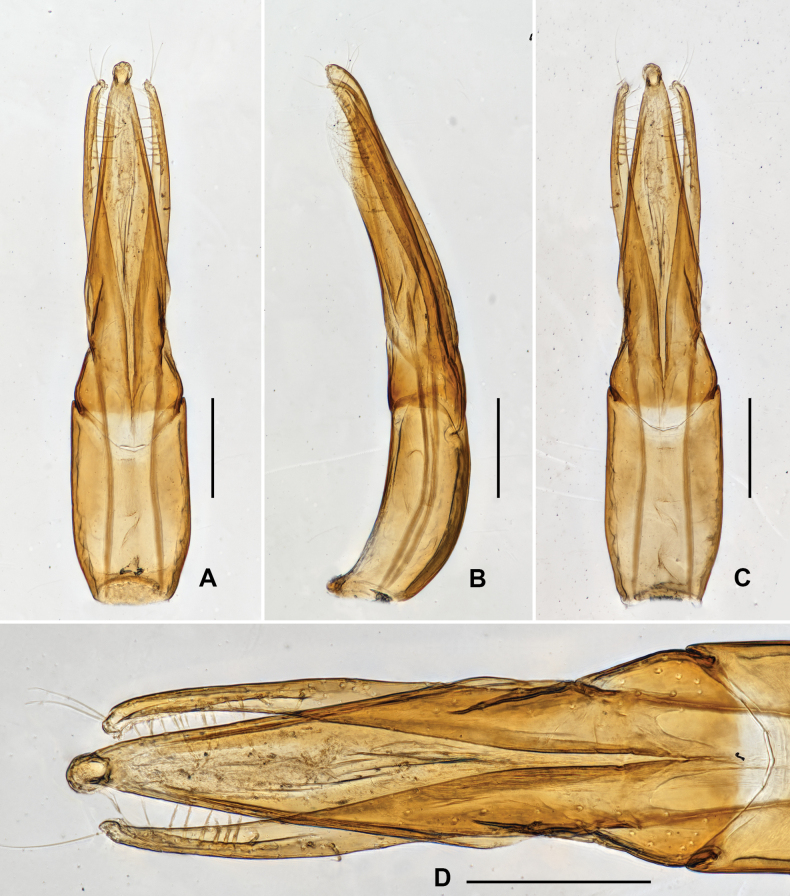
Aedeagus of *Grouvellinuswangmoensis* sp. nov., paratype **A v**entral view **B** lateral view **C** dorsal view **D** apex of median lobe and parameres. Scale bars: 0.05 mm (**D**); 0.1 mm (**A–C**).

Figs 1C , 6 , 7 , 12C, H

#### Material examined.

(31 exs: 11 ♂♂, 20 ♀♀): ***Holotype*: China** • ♂, labeled ‘China: Guizhou, Qiannan Buyi and Miao Autonomous Prefecture (黔南布依族苗族自治州), Wangmo County (望谟县), Mashan Town (麻山镇), Kafa Village (卡法村), H: ~ 857 m, 10.VII.2022, Jiang Ri-Xin leg.’ (GUGC). ***Paratypes*: China** • 10 ♂♂, 20 ♀♀, with same label data as the holotype (GUGC).

#### Diagnosis.

Body long-oval, dark brown, with weak cupreous metallic luster. Pronotum widest at base, surface finely covered with small punctures, portions of anterior and posterior angles distinctly wrinkled. Base of pronotum with a pair of round foveae located at middle. Elytron interval VIII carinated. Median sulcus of metaventrite thin but distinct, extending from base to 3/4 length of metaventrite. Aedeagus with median lobe slightly longer than parameres, finely narrowed from basal 1/3 to apex, basal 1/6 distinctly narrowed, apex rounded and weakly expanded. Parameres of aedeagus very thin, weakly curved, apex rounded, lateral portion with long setae at apical 1/3, apex with several much longer setae.

#### Description.

**Male.** Body elongate-oval (Fig. 1C), dark brown with cupreous metallic luster, tibiae, antennae, and elytra light brown. Plastron area confined to head, except for frons, vertex and clypeus, lateral portions of prosternum, ninth elytral interval and epipleura, lateral portions of mesosternum, metaventrite, abdomen, and surfaces of femora.

Head (Fig. 6A) wider than long, dorsal surface shiny, densely covered with short setae (except discal part) and with sparse large punctures, each bearing a longer seta. Surface of clypeus shiny, with sparse small punctures and each bearing a long seta. Labrum transverse, approximately as long as clypeus, surface shiny, basal 1/2 hairless, apical 1/2 with sparse short setae, lateral margins with long bristles, apical margin rounded.

Pronotum (Fig. 6B), slightly wider than long, widest at base, narrowed anteriad. Surface shiny, disc finely with sparse small punctures and long setae, surface of anterior and posterior angles distinctly wrinkled. Longitudinal impression absent, with a pair of small round foveae at middle of pronotal base. Anterior margin arcuate, anterior angles distinctly produced and acutangular. Lateral margins finely curved. Basal margin trisinuate, emarginated before scutellum, posterior angles acutangular. Sublateral carinae short, ~ 1/5 length of pronotum, each side of carinae with a shallow and indistinct oblique impression, not touch the apex of sublateral carinae, extending from basal 1/3 of pronotum to near anterior angle. Prosternal process (Fig. 6D) with apex weakly rounded, surface distinctly wrinkled sparsely covered with large punctures and long setae.

Scutellum (Fig. 6B) widely triangular, approximately as long as wide, widest at base; surface weakly wrinkled, with several large punctures, each bearing seta which in different length. Lateral margins finely curved, apex obtuse.

Elytra (Fig. 6C) ~ 1.5 × as long as wide, widest at base, narrowed to apex. Surface lighter in color than head and pronotum, shiny, with cupreous metallic luster and rows of sparse long setae. Strial punctures large in basal 2/3, separated by ~ 1.5 × diameters, and much smaller and widely separated in apical 1/3. Granulated carina on interval VIII, other intervals flat. Hind wings well developed.

Metaventrite (Fig. 6D), surface rough and finely with long setae and large round punctures at disc. Median sulcus distinct, extending from posterior margin to 3/4 length of metaventrite. A pair of small round impression located at basal sides of median sulcus. Lateral sides of disc with series of elliptical shallow impression.

***Abdomen*.** Base of ventrite I (Fig. 6D) weakly wrinkled, middle portions of ventrites I–IV and anterior part of ventrite V shiny (Fig. 6D), finely covered with large punctures, each puncture bearing a long seta. Other parts of abdomen (Fig. 6D) with plastron and mixed with sparse long setae. Apex of ventrite V weakly curved. Ventrite I with a pair of well-developed admedian carinae, curved at middle, extending from base to apex.

Legs simple, surface granulated; femora widened, surface with plastron; inner side of tibiae with cleaning fringes; tarsi slightly shorter tibiae; tarsal claws simple.

***Genitalia*.** Aedeagus (Fig. 7A–D), long and slender, symmetrical; median lobe slightly longer than parameres, distinctly narrowed at basal 1/5, apical 4/5 finely get narrowed from base to apex, with apex rounded. Parameres (Fig. 7D) thin, weakly curved to inner side, with apex rounded; apical 1/2 with long setae, apex of paramere with several much longer setae.

**Female.** Externally similar to the male, averagely larger. Ovipositor as in Fig. 12C, H. Stylus short and narrow, very weakly curved at base, ~ 1/4 as long as distal portion of coxite. Coxite long, apex distinctly expanded, roundly broadened at outer margin, and with several short and curved sensilla. Distal portion of coxite nearly straight, surface finely covered with very short and acute setae. Proximal portion of coxite short, ~ 1/2 the length of distal portion, surface finely covered with very short and acute setae. Valvifers longer than coxite, longitudinal baculum curved.

#### Measurements.

**Male** (*n* = 10): CL: 2.10–2.23 mm (2.18±0.04); PL: 0.60–0.62 mm (0.62±0.01), PW: 0.73–0.82 mm (0.76±0.03); EL: 1.49–1.61 mm (1.56±0.03), EW: 0.95–1.04 mm (0.99±0.03).

**Female**: CL (*n* = 10): 2.20–2.27 mm (2.24±0.02); PL: 0.56–0.64 mm (0.61±0.03), PW: 0.73–0.78 mm (0.76±0.02); EL: 1.61–1.63 mm (1.62±0.01), EW: 1.02–1.04 mm (1.03±0.01).

#### Distribution.

China: Guizhou.

#### Biology.

All adults were collected from submerged stone in small ravine stream (Fig. 15F).

#### Etymology.

The specific epithet refers to the type locality: Wangmo County (Guizhou, China); the name is treated as an adjective.

#### Comparative diagnosis.

The new species is similar to *Grouvellinuspilosus* Jeng & Yang, 1998, *G.huaxiensis* Jiang, Huang & Chen, 2023, *G.muyinlini* sp. nov., and *G.lihaitaoi* sp. nov. in habitus. *Grouvellinuswangmoensis* sp. nov. can be distinguished from *G.pilosus* by the following characters: 1) base of pronotum with a pair of rounded foveae in middle (vs with a pair of elongate oval impressions), 2) granulated carinae present on elytron strial interval VIII (vs granulated carinae present on elytron strial interval VII). The new species can be easily distinguished from the other three species mentioned above by the sublateral carinae of pronotum being very short, not in contact with the oblique impression, and by the thin slender parameres of the aedeagus.

### ﻿ Grouvellinus lihaitaoi sp. nov.

Taxon classificationAnimaliaColeopteraElmidae

B9483348-9A35-508F-A89E-9CA16A2CCE3E

https://zoobank.org/6C4113AA-FCAF-44D4-845C-F3000D582D0A

**Figure 8.**
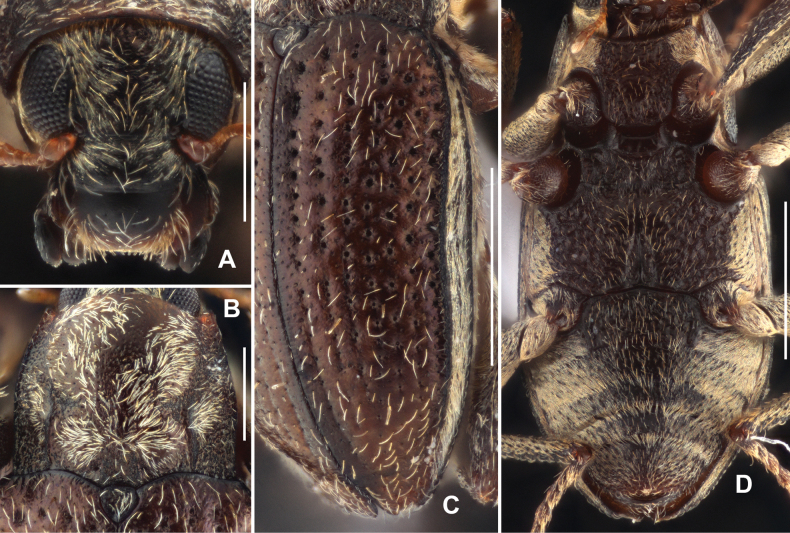
Diagnostic features of *Grouvellinuslihaitaoi* sp. nov., paratype **A** head, dorsal view **B** pronotum, dorsal view **C** elytra, dorsal view **D** ventral habitus. Scale bars: 0.25 mm (**A, B**); 0.5 mm (**C, D**).

**Figure 9.**
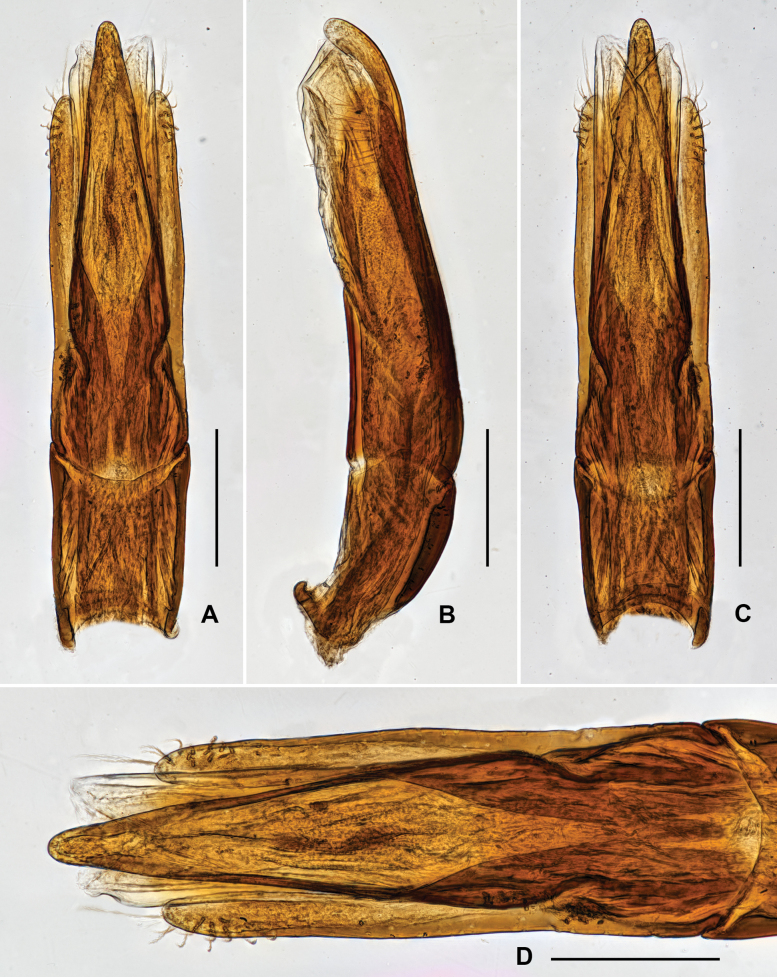
Aedeagus of *Grouvellinuslihaitaoi* sp. nov., paratype **A** dorsal view **B** same, lateral view **C** same, ventral view **D** apex of median lobe and parameres. Scale bars: 0.05 mm (**D**); 0.1 mm (**A–C**).

Figs 1D , 8 , 9 , 12D, I

#### Material examined.

(82 exs: 19 ♂♂, 13 ♀♀: 50 exs., sex undetermined): ***Holotype*: China** • ♂, labeled ‘China: Guizhou, Qiannan Buyi and Miao Autonomous Prefecture (黔南布依族苗族自治州), Longli (龙里县), Wantanhe Town (湾滩河镇), H: 1136.10±1.08m, 26°12'52"N, 106°59'27"E, 31.VIII.2023, Jiang Ri-Xin, Hai-Tao Li, Pin Li, Yu-Hao Zhang, Yin-Lin Mu & Xiu-Dong Huang leg.’ (GUGC). ***Paratypes*: China** • 18 ♂♂, 13 ♀♀, 50 exs., sex undetermined, with same label data as the holotype (GUGC).

#### Diagnosis.

Body elongate-oval, dark brown, shiny, with weak cupreous metallic luster. Pronotum widest at base, disc covered with dense small round punctures, surface of posterior angles granulated, middle of base a pair of small round foveae. Elytral interval VIII carinated, carina granulated. Median sulcus of metaventrite thin but distinct, extending from base to 1/2 length of mataventrite. Aedeagus with median lobe distinctly longer than parameres, base distinctly narrowed, apex narrowed, subacute. Parameres wide, apex rounded, outer sides weakly sinuate, lateral portion with long setae at apical 1/6.

#### Description.

**Male.** Body long-oval (Fig. 1D), head, pronotum and femora dark brown, elytra, tibiae, and antennae reddish brown, surface shiny with cupreous metallic luster. Plastron area confined to head, except for frons, vertex, and clypeus, lateral portions of prosternum, ninth elytral interval and epipleura, lateral portions of mesosternum, metaventrite, abdomen, and surfaces of femora.

Head (Fig. 8A) wider than long, dorsal surface shiny, densely covered with short setae (except discal part) and sparse large punctures, each puncture bearing a longer seta. Clypeus with surface shiny, densely covered with short setae (except disc) and sparse long setae. Labrum transverse, narrower than clypeus, surface shiny, basal 1/2 hairless, apical 1/2 with sparse short setae, lateral margins with long bristles, apical margin rounded.

Pronotum (Fig. 8B), slightly wider than long, widest at base, narrowed anteriad. Surface shiny, disc densely punctured, and with punctures in different length, intervals glabrous, shiny; longitudinal impression absent, with a pair of small round foveae located at median of base; surface of posterior angles distinctly granulated. Anterior margin arcuate, anterior angles produced and weakly acutangular with subacute apex; pronotal margins finely curved. Basal margin trisinuate, posterior angles acutangular. Sublateral carinae distinct, extending from base to ~ 2/5 length of pronotum, each side of sublateral carinae with a shallow and indistinct oblique impression, extending from apex of sublateral carinae to near anterior angle. Prosternal process (Fig. 8D) with apex rounded, surface distinctly wrinkled and sparsely covered with large punctures and long setae.

Scutellum (Fig. 8B) widely triangular, approximately as long as wide, widest at basal 1/3; surface weakly wrinkled, sparsely covered with large punctures, each bearing a long seta. Lateral margins finely curved, apex obtuse.

Elytra (Fig. 8C) ~ 1.5 × as long as wide, widest near apical 1/3. Surface shiny, with sparse long setae. Lateral margins sub-parallel in basal 2/3. Strial punctures larger in basal 2/3, separated by more than twice diameter, and much smaller and widely separated in other portions of elytra. Granulated carina on interval VIII, other intervals flat. Hind wings well developed.

Metaventrite (Fig. 8D), surface rough and finely with long setae and large round punctures at disc. Median sulcus distinct, extending from posterior margin to 1/2 length of metaventrite, base with a pair of round impression. Lateral sides of disc with series of elliptical shallow impression.

#### *Abdomen*.

Base of ventrite I (Fig. 8D) weakly wrinkled, middle regions of ventrites I–IV and anterior part of ventrite V (Fig. 8D) finely with large round punctures, each puncture bearing a long seta. Other parts of abdomen (Fig. 8D) covered with plastron and mixed with sparse long setae. Apex of ventrite V weakly curved, ventrite I with well-developed pair of admedian carinae, curved at middle, extending from base to apex.

Legs simple, surface granulated; femora widened, surface with plastron; inner side of tibiae with cleaning fringes; tarsi slightly shorter tibiae; tarsal claws simple.

#### *Genitalia*.

Aedeagus (Fig. 9A–D), long and slender, symmetrical; median lobe distinctly longer than parameres, widest at basal 2/5, apical 3/5 finely narrowed, with apex rounded. Parameres (Fig. 9D) wide, nearly straight, with apex rounded; apical 1/6 covered with long setae.

**Female.** Externally similar to the male, averagely larger. Ovipositor as in Fig. 12D, I. Stylus short and narrow, weakly curved near middle, ~ 1/4 as long as distal portion of coxite. Coxite long, with apex expanded, roundly broadened at outer margin, with several short and curved sensilla. Distal portion of coxite nearly straight, surface finely covered with very short and acute setae. Proximal portion of coxite short, longer than 1/2 the length of distal portion, surface finely covered with very short and acute setae. Valvifers slightly longer than coxite, longitudinal baculum weakly curved.

#### Measurements.

**Male** (*n* = 10): CL: 1.77–2.13 mm (1.88±0.10); PL: 0.49–0.61 mm (0.55±0.04), PW: 0.61–0.77 mm (0.69±0.05); EL: 1.28–1.51 mm (1.38±0.06), EW: 0.78–0.98 mm (0.87±0.06).

**Female**: CL (*n* = 10): 1.87–1.99 mm (1.93±0.05); PL: 0.52–0.54 mm (0.53±0.01), PW: 0.64–0.67 mm (0.65±0.01); EL: 1.27–1.44 mm (1.38±0.06), EW: 0.84–0.92 mm (0.88±0.03).

#### Distribution.

China: Guizhou.

#### Biology.

All adults were collected from submerged stone in small ravine stream (Fig. 15C–E).

#### Etymology.

The species epithet honors our friend and colleague Dr. Hai-Tao Li (Guizhou University), one of the collectors of the new species; the name is treated as an adjective.

#### Comparative diagnosis.

The new species is most similar to *Grouvellinusmuyinlini* sp. nov. It can be distinguished from that species by the following characters: pronotum widest at base (vs widest near middle); median sulcus of metaventrite short, ~ 1/2 the length of metaventrite (vs much longer, ~ 3/4 the length of metaventrite); parameres of aedeagus much wider, apex nearly straight (vs parameres much narrower, apex curved).

### ﻿ Grouvellinus muyinlini sp. nov.

Taxon classificationAnimaliaColeopteraElmidae

37748DEA-A1ED-5C01-A806-6318640433A3

https://zoobank.org/84E521A3-CEA7-4C73-9909-B8CAE775C711

**Figure 10.**
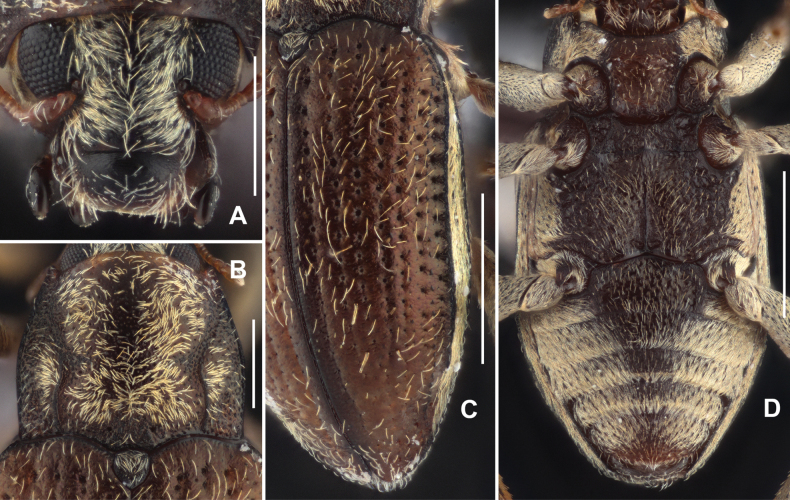
Diagnostic features of *Grouvellinusmuyinlini* sp. nov., paratype **A** head, dorsal view **B** pronotum, dorsal view **C** elytra, dorsal view **D** ventral habitus. Scale bars: 0.25 mm (**A, B**); 0.5 mm (**C, D**).

**Figure 11.**
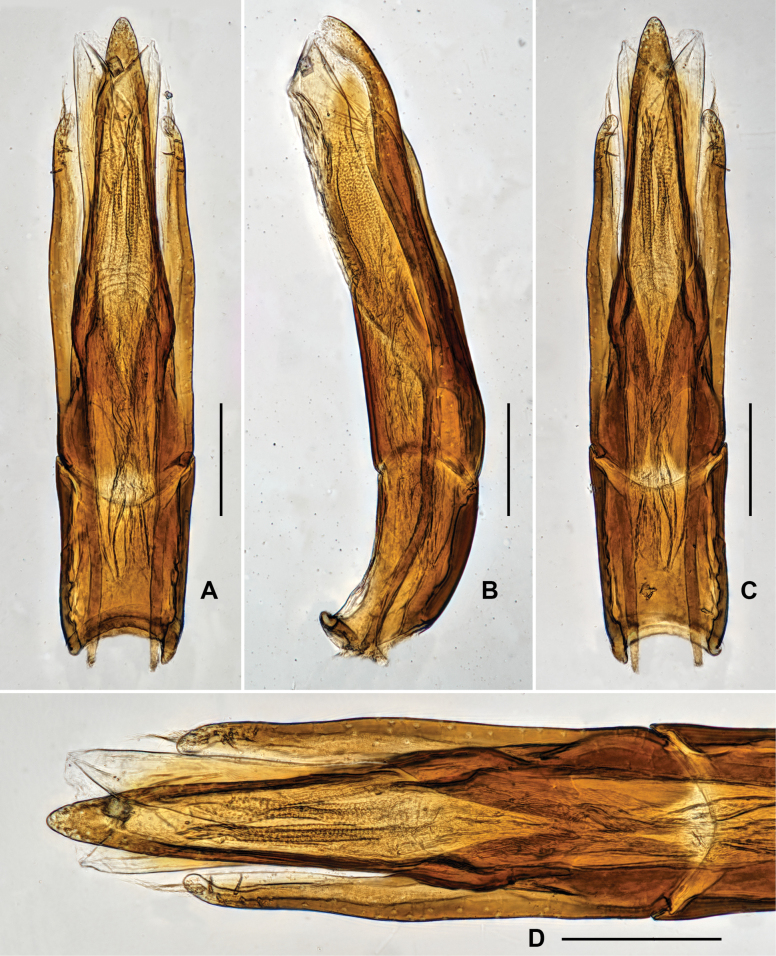
Aedeagus of *Grouvellinusmuyinlini* sp. nov., paratype **A** ventral view **B** lateral view **C** dorsal view **D** apex of median lobe and parameres. Scale bars: 0.05 mm (**D**); 0.1 mm (**A–C**).

**Figure 12.**
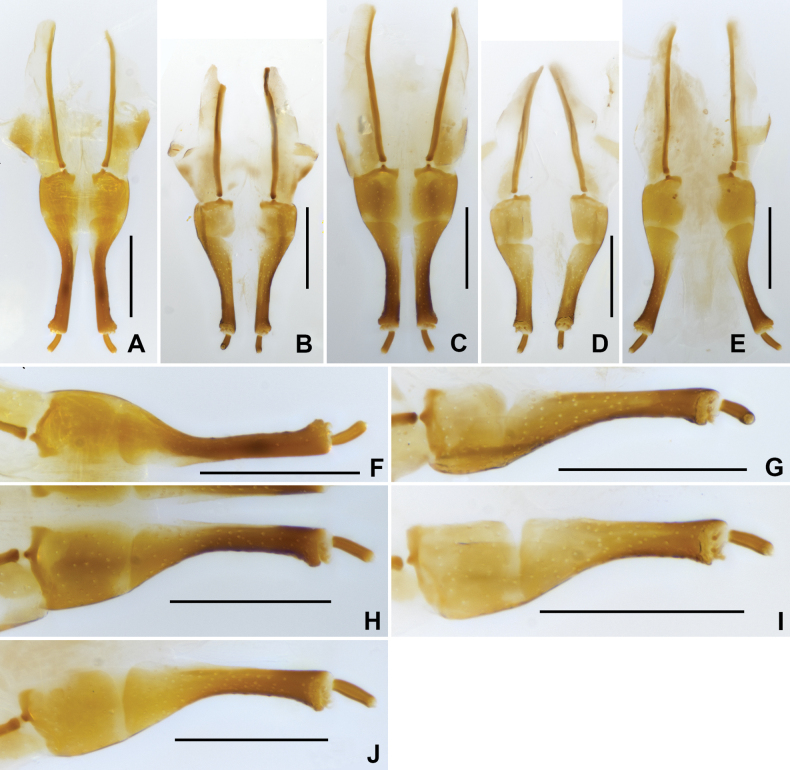
Ovipositor of *Grouvellinus* species **A, F***G.loong* sp. nov., paratype **B, G***G.buyi* sp. nov., paratype **C, H***G.wangmoensis* sp. nov., paratype **D, I***G.lihaitaoi* sp. nov., paratype **E, J***G.muyinlini* sp. nov., paratype. Scale bars: 0.1 mm.

Figs 1E , 10 , 11 , 12E, J

#### Material examined.

(40 exs: 20 ♂♂, 20 ♀♀): ***Holotype*: China** • ♂, labeled ‘China: Guizhou, Guiyang City (贵阳市), Huaxi District (花溪区), Qiantao Buyi and Miao Township (黔陶布依族苗族自治乡), Machang Village (马场村), Raolongxiagu (绕拢峡谷), H: 1084 m, 26°19'12"N, 106°46'19"E, 13.VII.2022, Jiang Ri-Xin, Yin-Lin Mu, Tian-Jun Liu & Feng-E Li leg.’ (GUGC). ***Paratypes*: China** • 19 ♂♂, 20 ♀♀, with same label data as the holotype (GUGC).

#### Diagnosis.

Body elongate-oval, dark brown with antenna, elytra and tibia pale brown, surface shiny with weak cupreous metallic luster. Elytral interval VIII carinated. Pronotum widest near middle, disc with dense small punctures, portions of posterior angles granulated, base of pronotum with a pair of median foveae. Median sulcus of metaventrite thin but distinct, extending from base to 3/4 length of metaventrite. Aedeagus with median lobe distinctly longer than parameres, constricted at base, finely narrowed from basal 1/5 to apex, apex rounded. Parameres thinner and shorter than median lobe, weakly sinuated and curved at apex, lateral portion with long setae at apical 1/6.

#### Description.

**Male.** Body elongate-oval (Fig. 1E), head and elytra dark brown with elytra, tibiae, antennae, and elytra pale brown, dorsal surface shiny with cupreous metallic luster. Plastron area confined to head except for frons, vertex, and clypeus, lateral portions of prosternum, ninth elytral interval and epipleura, lateral portions of mesosternum, metaventrite, abdomen, and femora.

Head (Fig. 10A) wider than long, dorsal surface shiny, densely covered in short setae (except discal part) and sparse longer setae. Clypeus shiny, with dense short setae (except disc) and sparse long setae. Labrum transverse, slightly narrower than clypeus, shiny, basal 1/2 hairless and microreticulated, apical 1/2 with sparse short setae, lateral margins with long bristles, apical margin rounded.

Pronotum (Fig. 10B), slightly wider than long, widest near middle. Disc with dense punctures in different length and long setae, intervals of punctures glabrous, longitudinal impression absent, with a pair of small round foveae located at middle base near posterior margin, surface of posterior angles granulated. Anterior margin arcuate, anterior angles produced and weakly acutangular. Lateral margins finely curved. Basal margin trisinuate, emarginated before scutellum, posterior angles acutangular. Sublateral carinae distinct, extending from base to ~ 2/5 length of pronotum, each side of sublateral carinae with a shallow and indistinct oblique impression, extending from apex of sublateral carinae to near anterior angle. Prosternal process (Fig. 10D) with apex rounded, surface wrinkled and sparsely covered with large punctures and long setae.

Scutellum (Fig. 10B) widely triangular, approximately as long as wide, widest near middle; surface densely punctured, each puncture bearing a long seta. Lateral margins finely curved, apex obtuse.

Elytra (Fig. 10C) widest near apical 3/7, reddish brown, lateral margins sub-parallel in basal 4/7. Surface shiny, with weakly cupreous metallic luster and sparse long setae. Granulate carinae on interval VIII, interval VII with a row of sparse and very small granules, extending from base to apex; other intervals flat. Strial punctures larger in basal 4/7, separated by more than 2 × diameter, and much smaller and widely separated in other part of elytra. Hind wings well developed.

Metaventrite (Fig. 10D), surface finely punctured with large punctures and long setae at disc, lateral sides of disc with series of elliptical shallow impression. Median sulcus distinct, extending from posterior margin to 3/4 length of metaventrite, with a pair of round impression at sides of basal median sulcus.

***Abdomen*.** Base of ventrite I (Fig. 10D) weakly wrinkled, middle regions of ventrites I–IV and anterior part of ventrite V (Fig. 10D) punctured with large and round punctures, each puncture bearing a long seta. Other parts of abdomen (Fig. 10D) with plastron and mixed with sparse long setae. Apex of ventrite V weakly curved. Ventrite I with a pair of well-developed admedian carinae, curved at middle, extending from base to apex.

Legs simple, surface granulated; femora widened, surface covered with sericeous tomentum; inner side of tibiae with cleaning fringes; tarsi slightly shorter than tibiae; tarsal claws simple.

***Genitalia*.** Aedeagus (Fig. 11A–D), long and wide, symmetrical; median lobe distinctly longer than parameres, constricted at base, finely narrowed from basal 1/5 to apex, with apex rounded. Parameres (Fig. 11D) thinner and shorter than median lobe, weakly sinuated and curved at apex, lateral portion with long setae at apical 1/6.

**Female.** Externally similar to the male, averagely larger. Ovipositor as in Fig. 12E, J. Stylus short and narrow, nearly straight, weakly expanded at middle, shorter than 1/4 length of distal portion of coxite. Coxite long, apex distinctly expanded, roundly broadened at outer margin, with several short and curved sensilla. Distal portion of coxite nearly straight, surface finely covered with very short and acute setae. Proximal portion of coxite short, ~ 1/2 the length of distal portion, surface finely covered with very short and acute setae which are sparser than setae on distal portion of coxite. Valvifers slightly longer than coxite, longitudinal baculum nearly straight, weakly curved at base.

**Figure 13.**
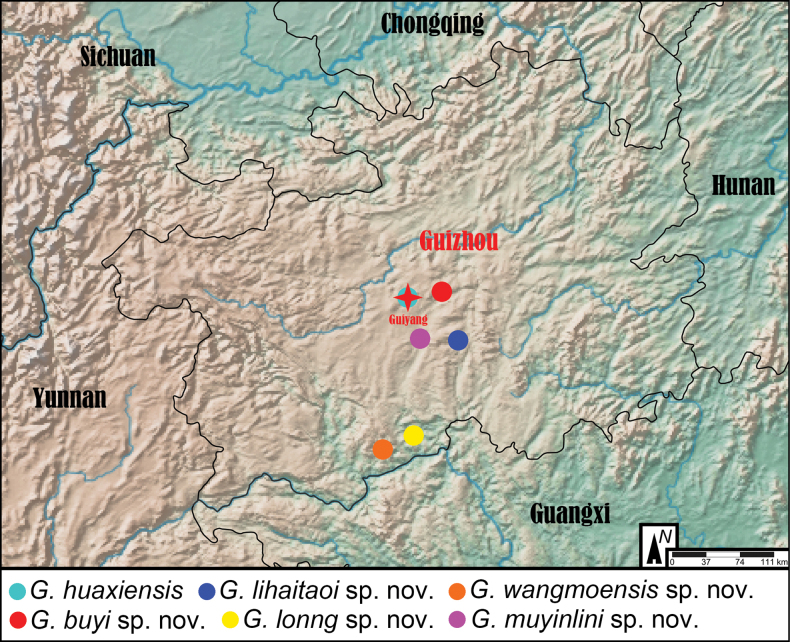
Distribution map of known *Grouvellinus* species from Guizhou Province.

**Figure 14.**
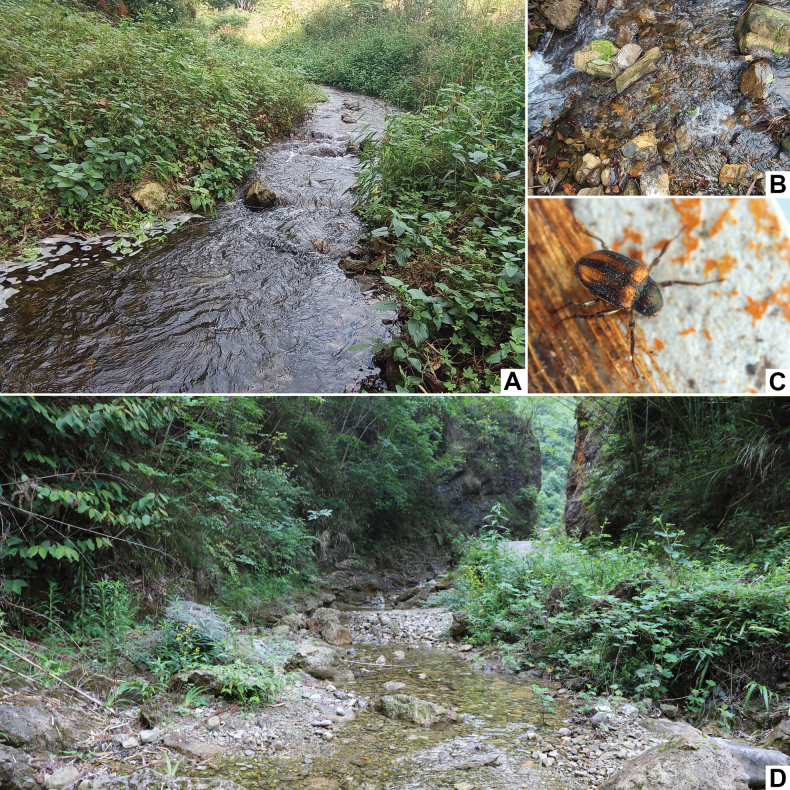
Habitats of *Grouvellinus* species **A** general environment of the type locality of *G.loong* sp. nov. **B** same, microenvironment **C** living adult of *G.loong* sp. nov. **D** general environment of the type locality of *G.buyi* sp. nov.

#### Measurements.

**Male** (*n* = 10): CL: 1.78–2.09 mm (1.89±0.09); PL: 0.49–0.58 mm (0.53±0.03), PW: 0.62–0.68 mm (0.65±0.02); EL: 1.26–1.52 mm (1.35±0.07), EW: 0.81–0.90 mm (0.86±0.03).

**Female** (*n* = 10): CL: 1.90–2.11 mm (2.00±0.07); PL: 0.55–0.59 mm (0.57±0.01), PW: 0.68–0.72 mm (0.70±0.01); EL: 1.36–1.52 mm (1.42±0.07), EW: 0.85–1.02 mm (0.92±0.05).

#### Distribution.

China: Guizhou.

#### Biology.

**Figure 15.**
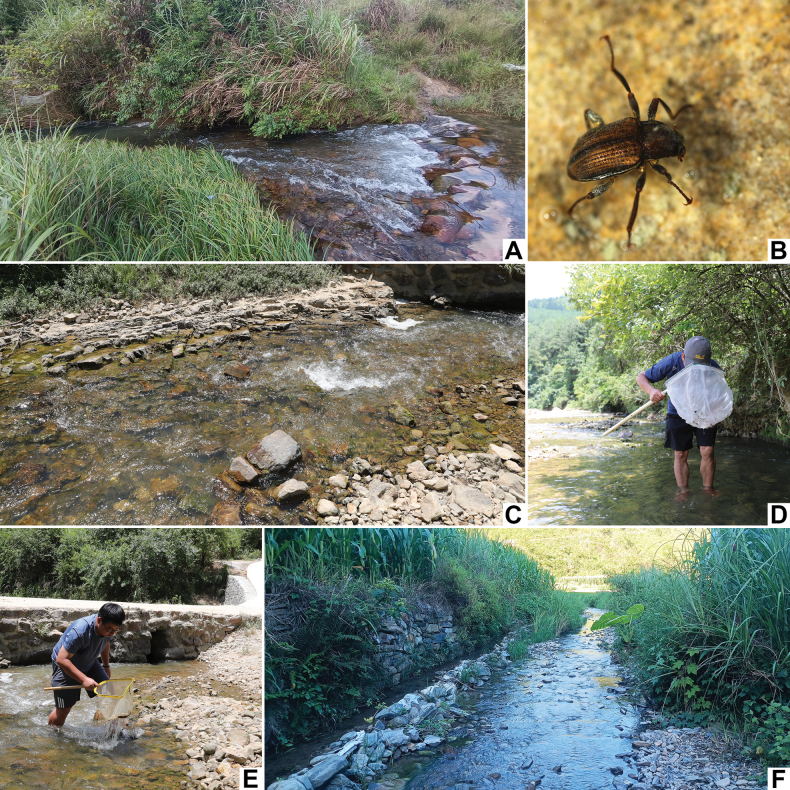
Habitat of *Grouvellinus* species **A** general environment of the type locality of *G.lihaitaoi* sp. nov. **B** living adult of *G.lihaitaoi* sp. nov. **C** general environment of *G.muyinlini* sp. nov. **D** dr. Yin-Lin Mu working in the type locality of *G.muyinlini* sp. nov. **E** the first author working in the same locality as ditto **F** general environment of *G.wangmoensis* sp. nov.

All adults were collected from submerged stone in small ravine stream (Fig. 15C–E).

#### Etymology.

The species epithet honors our friend and colleague Dr. Yin-Lin Mu (Guizhou University), one of the collectors of the new species; the name is treated as an adjective.

#### Comparative diagnosis.

*Grouvellinusmuyinlini* sp. nov. is most similar to *G.lihaitaoi* sp. nov. For differences see the comparative diagnosis of *G.lihaitaoi* sp. nov.

## ﻿Discussion

Recent papers suggested that the genus *Grouvellinus* has a potentially high diversity in Southeast Asia, and particularly in South China (Bian and Sun 2016; Bian and Jäch 2018, 2019; Freitag et al. 2018, 2020; Bian and Zhang 2023; Jiang et al. 2023). Freitag et al. (2020) reported a surprising diversity of *Grouvellinus* in a small geographic range of Sabah, and a similar situation can also be found in Guizhou Province, China, where several (three or four) species were collected. On the other hand, some species show stronger adaptation to poor water quality, e.g., *G.huaxiensis* Jiang, Huang & Chen, 2023 was collected in an urban river of Guiyang City.

Some *Grouvellinus* species are highly similar in habitus. However, a simple clustering combined with morphological study is helpful to effectively distinguish species of this group. Moreover, the true diversity of this group in China still needs to be unveiled, especially in the mountain and karst areas of South China.

## Supplementary Material

XML Treatment for Grouvellinus

XML Treatment for Grouvellinus loong

XML Treatment for Grouvellinus buyi

XML Treatment for Grouvellinus wangmoensis

XML Treatment for Grouvellinus lihaitaoi

XML Treatment for Grouvellinus muyinlini

## ﻿Additional information

### Author ORCIDs

Ri-Xin Jiang https://orcid.org/0000-0002-5339-853X

Ping-Lan Wu https://orcid.org/0009-0003-7089-7861

Xiang-Sheng Chen https://orcid.org/0000-0001-9801-0343

### Data availability

All of the data that support the findings of this study are available in the main text.
